# The Effect of Myopic Control between the Dual-Focus Contact Lenses and High-Concentration Atropine in an Asian Population

**DOI:** 10.3390/life14010118

**Published:** 2024-01-13

**Authors:** Chia-Yi Lee, Shun-Fa Yang, Yu-Ling Chang, Jing-Yang Huang, Ie-Bin Lian, Chao-Kai Chang

**Affiliations:** 1 Institute of Medicine, Chung Shan Medical University, Taichung 402, Taiwan; 2 Nobel Eye Institute, Taipei 115, Taiwan; 3 Department of Ophthalmology, Jen-Ai Hospital Dali Branch, Taichung 412, Taiwan; 4 Department of Medical Research, Chung Shan Medical University Hospital, Taichung 402, Taiwan; 5 Department of Medical Education, Cathay General Hospital, Taipei 106, Taiwan; 6 Institute of Statistical and Information Science, National Changhua University of Education, Changhua 500, Taiwan; 7 Department of Optometry, Da-Yeh University, Chunghua 515, Taiwan

**Keywords:** atropine, dual-focus contact lenses, spherical equivalent refraction, axial length, astigmatism

## Abstract

We aim to investigate the myopic control effect of high-concentration atropine (ATR) and dual-focus contact lenses (DFCLs). A retrospective cohort study was conducted. A total of 182 eyes in 91 individuals who used high-concentration ATR (0.125%) and another 70 eyes in 35 individuals who used DFCLs were enrolled in the ATR and DFCL groups, respectively. The primary outcomes were spherical equivalent refraction (SER) progression and axial length (AXL) elongation. The generalized estimate equation was utilized to yield the adjusted odds ratio (aOR) and 95% confidence interval (CI) of cycloplegic SER progression and AXL elongation between groups. According to the multivariable analysis, the change in cycloplegic SER progression was similar between the DFCL and ATR groups (aOR: 1.305, 95% CI: 0.247–2.515, *p* = 0.803). The DFCL group demonstrated a numerically higher rate of AXL elongation compared to the ATR group (aOR: 1.530, 95% CI: 0.980–1.894, *p* = 0.051). In the subgroup analysis, cycloplegic SER progression was insignificant between ATR and DFCL users in different subgroups (all *p* > 0.05). The DFCL patients with moderate astigmatism and high AXL (both *p* < 0.001) presented a high risk of AXL elongation. In conclusion, DFCL usage demonstrated similar myopic control of cycloplegic SER and AXL compared to high-concentration ATR, while DFCLs showed lower AXL control, mainly in patients with moderate astigmatism and high AXL.

## 1. Introduction

Myopia, which has recently shown increased occurrence, is a disease in which images focus in front of the retinal plane, causing blurry vision [1,2]. Normally, rays enter the eye and hit the center of the retina, and the photoreceptors on the retina collect the light signals. Then, the light signals are converted to electrical signals and pass through the retina to reach the optic nerve. The optic nerve then transports these signals into the occipital lobe, where visual signals are managed, producing vision. In patients with myopia, light enters the eye and focuses on a spot in front of the retina, and the photoreceptors cannot produce sufficient signals, thus causing blurry vision.

The etiologies of the formation of myopia, or the reasons for light focusing in front of the retina rather than on the retina, are axial myopia and refractive myopia [1,2]. Axial myopia is caused by the eyeball growing too long compared to the eye’s refractive power, which is the most common type of childhood/acquired myopia. The axial length of the eyeball is the distance from the cornea to the retina, and light will focus in front of the retina rather than directly hit it if the eyeball is too long, causing distant images to appear blurry [1,2]. In comparison, refractive myopia develops if the curve of the cornea or the crystalline lens is extremely steep. The prevalence of refractive myopia is not as common as axial myopia [1,2]. In rare episodes, refractive myopia can also occur if the lens is located too close to the cornea. It is also possible to possess myopia that combines both the axial and refractive pathophysiologies. 

Concerning the rate of myopia, the annual prevalence of myopia is nearly 44 percent in the Caucasian population and about 80 percent in the Eastern Asian region due to differences in lifestyle [3]. The main etiologies for the development of myopia include the steepening of the corneal curvature and the elongation of the eyeball, the latter of which tends to progress after birth [4]. High myopia, which is regarded as a sphere power over −5.00 diopter (D), contributes to higher rates of optic nerve damage and myopic maculopathy, and the importance of preventing the development of high myopia cannot be overemphasized [5].

Different methods of myopia control have previously been evaluated and applied [1,6]. Atropine (ATR) is a medication used for cycloplegic refraction, or dilating the pupil, for the examination of the retina and the execution of cataract and retinal surgery [2,7], and the application of ATR in children can also slow myopia progression. The usage of ATR for myopia control can be traced back to the 1990s when no spectacle-related myopia-control products were available. The mechanism of ATR’s myopia control effect is due to the retardation of axial length elongation rather than the cycloplegic function that relieves the pupil sphincter [2,7]. The application of high-concentration ATR reduces the degree of myopia progression effectively, but complications, including photophobia, blurry vision at reading distance, and ocular allergies, may occur [2,7]. The elevation of intraocular pressure after ATR utilization has been reported, but its incidence is extremely rare, and thus, the safety of ATR can be guaranteed with regard to ocular hypertension and subsequent glaucoma [2,7].

In addition, orthokeratology contact lenses can contribute to a good myopia control rate with regard to refractive status [8,9]. The orthokeratology contact lens was first proposed in the 1960s in which the initial orthokeratology contact lens resembled a rigid gas-permeable contact lens. However, the visual outcome and refractive predictability of the initial orthokeratology contact lenses were poor, and they were soon abandoned. The re-introduction of orthokeratology contact lenses occurred in the 1990s, which included the following three innovations: the reverse-geometry shape design of orthokeratology contact lenses, the emergence of high-oxygen-permeability material, and the clinical application of topography in the production of orthokeratology contact lenses [8,9]. Subsequently, orthokeratology contact lenses became a credible and effective tool for controlling myopic progression. Moreover, nearly all patients with orthokeratology contact lenses achieve spectacle independence, elevating daily convenience without a reduction in visual quality [10]. In fact, the visual quality of children wearing the orthokeratology contact lenses was shown to be numerically better than those wearing spectacle lenses [10].

When comparing the effects of ATR and orthokeratology contact lenses on myopia control, spherical equivalent refraction (SER) progression and axial length (AXL) elongation were shown to be similar between high-concentration ATR and orthokeratology contact lenses [11]. In terms of complications, irritation was observed in both the ATR and orthokeratology contact lens groups, although its severity was not enough to discard the treatments altogether [11]. Consequently, we can say that both ATR and orthokeratology contact lenses are safe and efficient tools for myopic control.

Except for the rigid design of orthokeratology contact lenses, several soft contact lenses have been produced for the purpose of controlling myopia [1,8,12,13]. Among them, dual-focus contact lenses (DFCLs) were first introduced in the mid-2010s and showed a fair myopic control effect concerning SER progression and AXL elongation compared to individuals using general soft contact lenses [14]. When considering side effects in individuals using DFCLs, only one patient was diagnosed with peripheral corneal haze, which did not interfere with visual function [14]. In addition, one patient developed uveitis during the application of DFCLs, but uveitis is less likely to be triggered by DFCLs, according to [14]. When comparing DFCLs and high-concentration ATR, previous studies have shown a numerically higher myopic control effect in high-concentration ATR [7,8,15]; however, these previous studies compared the effect of DFCLs and high-concentration ATR using different study designs and regions [7,8,15], and a comparison between DFCLs and high-concentration ATR using similar conditions has not been conducted.

Thus, the aim of the current study is to compare the myopic control effects of high-concentration ATR and DFCLs on the management of SER progression and AXL elongation in the individuals receiving these treatments. All the patients enrolled in the current study resided in the Taiwan region.

## 2. Materials and Methods

### 2.1. Participant Selection

A retrospective cohort study was performed at the Nobel Eye Institute from 1 October 2020 to 31 September 2022. The Nobel Eye Institute is a joint clinic group that owns several branches in the northern, central, and southern regions of Taiwan. The inclusion criteria of our participants were (1) age younger than 15 years, (2) use of high-concentration ATR or DFCLs in our clinics, and (3) regular follow-ups in any branch of our clinic for at least one year with a minimum of six appointments per year. One brand of high-concentration ATR (0.125% Tropine, Aseptic Innovative Medicine Co., Ltd., Taoyuan Dist, Taoyuan City, Taiwan) and one brand of DFCLs (MiSight, CooperVision, Victor, NY, USA) were used in our study. To choose the myopic control tool, the ophthalmologist proposed all types of myopic control methods, if no absolute contraindications existed (i.e., a 5-year-old child for DFCL usage), to the children and parents. Then, the ophthalmologist discussed the selection of the myopic control method with the parents (and potentially also the children), where different ophthalmologists’ habits and opinions of each myopic control tool could influence the selection result. Finally, the parents chose the type of myopic control method for the children, and the ophthalmologist prescribed the tool for them if no absolute contraindication was present. In our clinics, DFCL users wore the contact lenses for 10 h per day, every day without breaks. The ATR group was given atropine between 21:00 and 23:00, just before sleep without breaks. Because this period is relatively far from the measurement intervals (15:00 to 21:00) used in our clinic, ATR instillation might not influence the SER measurement to a large extent. In addition, several exclusion criteria were applied to decrease the heterogeneity of the study population and exclude extreme visual impairment: (1) baseline best-corrected visual acuity (BCVA) lower than 20/40 on a Snellen chart, (2) high myopia with sphere power higher than −5.00 D, (3) high astigmatism with cylinder power higher than −2.50 D and (4) severe ocular defects including but not limited to corneal scars, corneal neovascularization, congenital glaucoma, congenital cataracts, advanced retinopathy of prematurity, optic nerve atrophy and color blindness. Additionally, individuals using DFCLs with any ATR application were also excluded. After the whole process, a total of 182 eyes in 91 patients and 70 eyes in 35 patients were categorized into the ATR group and DFCL group, respectively.

### 2.2. Primary Outcome

The basic characteristics of each individual, including age, sex, pre-treatment BCVA, sphere power, cylinder power, and AXL, were obtained from the medical documents. The BCVA was performed with a trial frame, and the optimal visual acuity was refined by our optometrists. The sphere power was tested first, followed by the cylinder axis. After that, the cylinder power was determined. With regard to the parameters of myopic progression, the primary outcomes in the current study were SER progression and AXL elongation during the one-year follow-up period. The refraction and AXL were examined with an autorefractor (KR-8900, Topcon, Itabashi-ku, Tokyo, Japan) and biometry device (IOL Master 500, Carl Zeiss, Göschwitzer Str., Jena, Germany) in each branch of our clinic. The autorefractor measurement was performed three times, and the optical biometry was performed once for each patient. Cycloplegic refraction was performed for all the participants before beginning myopic control management and about one year after myopic control management. Refraction, including sphere and cylinder, was measured three times; the average values of the three results were entered into the dataset of the current study, and the sphere power plus half of the cylinder power was defined as the SER. All the optometry rooms in our clinics adhere to the law of optometrists in Taiwan; the room sizes, devices, light conditions, and distances between the Snellen chart and patient are the same among all the branches of our clinic. Also, the brands of Snellen chart and optical biometry are the same among all the branches of our clinic, and the time of measurement usually ranges from 15:00 to 21:00. Both SER and AXL values before management, 1 month after management, 3 months after management, 6 months after management, 9 months after management, and 12 months after management in the two groups were recorded and used in the following statistical analysis.

### 2.3. Statistical Analysis

SPSS version 20.0 (SPSS Inc., Chicago, IL, USA) was used for the statistical analyses of the current study. We used descriptive analysis to illustrate the basic characteristics between the two groups, and the Chi-square test and independent T-test were applied for the comparison of basic characteristics between the two groups depending on the parameters. After that, the independent T-test was executed to compare the baseline cycloplegic SER and AXL and final cycloplegic SER and AXL between the two groups, and the generalized estimate equation was adopted for analyzing the alteration of cycloplegic SER progression and AXL elongation between the two populations with adjustment of the effects of sex, age, pre-treatment BCVA, pre-treatment AXL, and pre-treatment cycloplegic SER. The adjusted odds ratio (aOR) with 95% confidence interval (CI) of the DFCL group compared to the ATR group was yielded by the generalized estimate equation. Also, we plotted a line chart for the trends of manifest SER progression and AXL elongation of the ATR and DFCL groups during the study interval. For the subgroup analysis, the total SER and AXL changes in individuals receiving high-concentration ATR or using DFCLs with moderate myopia (more than −3.00 D), moderate astigmatism (more than −1.50 D), high AXL (more than 25.00 mm) and old age (older than 12 years old) were evaluated separately using the generalized estimate equation. The statistical significance was defined as *p* < 0.05 in the current study, and a *p*-value lower than 0.001 was described as *p* < 0.001.

## 3. Results

### 3.1. Basic Characteristics of the Study Population

The basic characteristics of the two groups are presented in Table 1. The mean age was 10.26 ± 2.56 and 12.33 ± 2.09 years old in the ATR and DFCL groups, and the DFCL group was significantly older than the ATR group (*p* < 0.001). Also, the DFCL group showed a higher baseline sphere power than the ATR group (−1.01 ± 1.42 versus −1.43 ± 1.47, *p* = 0.0028) (Table 1). The other parameters, including sex, baseline BCVA, and baseline cylinder power, were similar between the two groups (all *p* > 0.05) (Table 1).

### 3.2. Change in Refraction and Axial Length after Treatments

**Figure 1:**
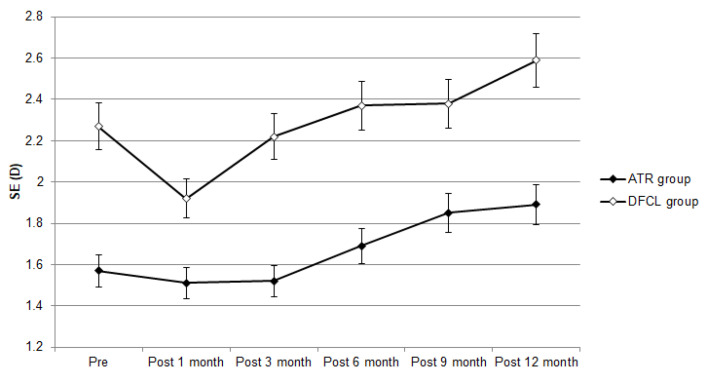
The trend of manifest spherical equivalent changes between the two groups. D: diopter, SE: spherical equivalent.

**Figure 2:**
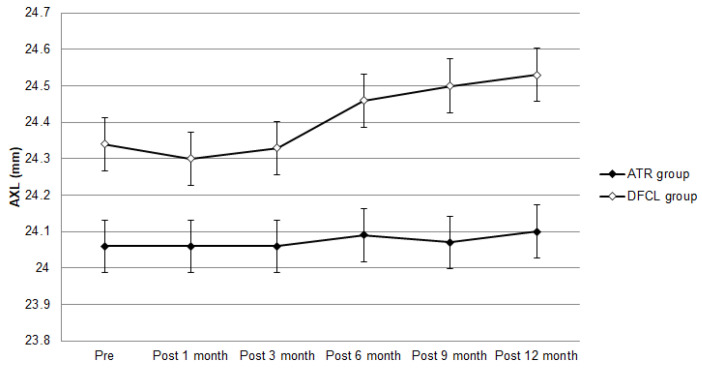
The trend of axial length changes between the two groups. AXL: axial length.

The baseline cycloplegic SER was −1.28 ± 1.41 D in the ATR group and −1.71 ± 1.56 D in the DFCL group, in which DFCL showed a higher initial cycloplegic SER (*p* < 0.001). After the follow-up interval, the cycloplegic SER was −1.59 ± 1.40 D in the ATR group and −2.03 ± 1.79 D in the DFCL group. The change in cycloplegic SER (−0.31 ± 0.87 versus −0.32 ± 0.89) was similar between the ATR and DFCL groups (aOR: 1.305, 95% CI: 0.247–2.515, *p* = 0.803) after adjusting for the effects of sex, age, pre-treatment BCVA, pre-treatment AXL and pre-treatment cycloplegic SER. On the other hand, the baseline AXL was 24.06 ± 0.72 mm and 24.34 ± 0.92 mm in the ATR and DFCL groups, respectively, and the DFCL group also showed a longer baseline AXL (*p* < 0.001). One year after the treatment, the AXL was 24.10 ± 0.78 mm in the ATR group and 24.53 ± 0.96 mm in the DFCL group (Table 2), and the DFCL group presented a significantly higher AXL after treatment compared to the ATR group (*p* = 0.007) (Table 2). However, the DFCL group only demonstrated a numerically higher rate of AXL elongation than the ATR group (aOR: 1.530, 95% CI: 0.980–1.894, *p* = 0.051) according to the result of the generalized estimate equation. The trends of manifest SER progression and AXL elongation are presented in Figure 1 and Figure 2.

### 3.3. Subgroup Analyses with Different Parameters

In total, there were 37 eyes with ATR usage and 16 eyes with DFCL usage in the moderate myopia population, 12 eyes with ATR usage and 5 eyes with DFCL usage in the moderate astigmatism population, 48 eyes with ATR usage and 22 eyes with DFCL usage in the high-AXL population and 54 eyes with ATR usage and 46 eyes with DFCL usage in the old age population. In the subgroup analysis, the cycloplegic SER progression was insignificant between ATR and DFCL users in all four different subgroups, including the moderate myopia subgroup, moderate astigmatism subgroup, high-AXL subgroup, and the old age subgroup (all *p* > 0.05) (Table 3). Concerning the AXL elongation, the DFCL patients with moderate astigmatism (aOR: 1.893, 95% CI: 1.252–2.557, *p* < 0.001) and high AXL (aOR: 1.994, 95% CI: 1.568–2.817, *p* < 0.001) presented a high risk of AXL elongation compared to the ATR patients under the same conditions, and the trend of AXL elongation demonstrated no difference between the ATR and DFCL users with moderate myopia or old age (both *p* < 0.05) (Table 3).

## 4. Discussion

Briefly, the current study showed a similar cycloplegic SER progression in the high-concentration ATR and DFCL groups after a follow-up period of one year. Moreover, the speed of AXL elongation was comparable in the participants using DFCLs and the participants using high-concentration ATR. Also, the DFCL users with moderate astigmatism or high AXL tended to develop higher AXL elongation compared to high-concentration ATR users with similar characteristics.

The patients with high-concentration ATR and DFCL applications demonstrated statistically the same cycloplegic SER progression in the current study. In a previous publication, the application of high-concentration 0.1% ATR contributed to a slower SER progression of −0.47 ± 0.91 D compared to −1.06 ± 0.61 D in the control group [16]. On the other hand, the use of DFCLs was also correlated with a 50% decrease in SER compared to the control group using single contact lenses in the previous literature [14]. Nevertheless, previous studies have only compared high-concentration ATR or DFCL users to non-treatment individuals, and the setting of each study was different, which leads to a direct comparison between ATR application and DFCL usage becoming difficult [14,16]. To our knowledge, a preliminary finding may be that the utilization of high-concentration ATR and DFCLs contribute to similar effects on myopia control concerning the rate of SER progression. Except for the similar patient populations between the ATR and DFCL groups in the current study, we adjusted the influence of several confounders for myopic progression, including age, initial refractive power, and initial AXL in the multivariable model [17]. Moreover, the patients using DFCLs and ATR concurrently were excluded from the current study, and thus, the effect of DFCLs on myopic progression was independent. The similar cycloplegic SER progression between the ATR and DFCL groups is contrary to previous publications in which the application of high-concentration ATR was associated with better myopic control than DFCLs [5,14,16]. A possible explanation is that confounders for myopic progression had not been adjusted for in previous studies. On the other hand, high-concentration ATR presented a numerically lower AXL elongation compared to the DFCL population in the current study. AXL is a precise parameter for myopic progression [18], and the results of the current study may indicate similar overall myopic control of ATR and DFCLs. Furthermore, the difference in AXL elongation was 0.15 mm between the ATR group and DFCL group in the current study. Accordingly, the difference in AXL elongation and related myopic progression between high-concentration ATR and DFCL may not be clinically significant.

In the subgroup analysis, the change in cycloplegic SER after the study interval was similar between the high-concentration ATR and DFCL populations in all the specific subgroups. This result is identical to the whole-group analysis between the high-concentration ATR and DFCL groups and indicates that high-concentration ATR and DFCL showed the same effect on cycloplegic SER control in all patients. On the contrary, the individuals with moderate astigmatism and high initial AXL presented a better AXL control with the usage of high-concentration ATR, while the efficiency of AXL control between high-concentration ATR and DFCL was identical in patients with moderate myopia and old age. There was little research reporting this finding. The design of the DFCLs used in the current study did not contain an astigmatism correction function [14,19]. Although low-degree astigmatism can be corrected by spherical contact lenses, according to the experience of orthokeratology contact lenses [20], patients with moderate astigmatism higher than −1.50 D usually require toric orthokeratology contact lenses to obtain a good fit and fair vision [21]. Since the myopic control method was decided by the parents of our patients, some children with moderate astigmatism still applied DFCLs for myopic control. Thus, we speculate that myopic individuals with moderate astigmatism would experience worse visual quality when utilizing DFCLs, which is a risk factor for myopic progression [8]. On the contrary, the individuals who used high-concentration ATR were always equipped with daily spectacles, and their visual quality may be better than that of the DFCL users. The higher AXL elongation of DFCL users with high initial AXL was also observed. Still, the DFCL users showed similar AXL control to high-concentration ATR users regardless of age and myopia degree, and thus, DFCLs may have an advantage in myopic control in those without high AXL and moderate astigmatism because ATR-related complications including photophobia and blurry reading can be avoided [22,23].

With regard to the efficiency of myopic control in our study, a previous publication showed an annual SER progression of −0.87 ± 0.52 D in high-concentration ATR users (0.5 percent) [22]. For the AXL aspect, the range of annual AXL elongation in high-concentration ATR was 0.23 mm to 0.35 mm in a meta-analysis [24]. In the current study, the cycloplegic SER progression and AXL elongation after one year of high-concentration ATR application were −0.31 ± 0.87 D and 0.04 ± 0.39 mm, respectively. Compared to previous studies [5,22,24,25], the effect of ATR-associated myopic control in the current study was acceptable. On the other hand, a previous publication demonstrated SER progression and AXL elongation of −0.51 D and 0.30 mm after a three-year period after DFCL usage [14], and the subsequent experiment of that study presented SER progression and AXL elongation of −0.52 D and 0.23 mm after a six-year interval [26]. The SER progression and AXL elongation were −0.32 ± 0.89 D and 0.19 ± 0.26 mm one year after the application of DFCLs in the current study, which is comparable to preceding studies [14,26]. The comparisons between the current study and previous studies in terms of SER progression and AXL elongation are demonstrated in Table 4. In addition, one study used the combined therapy of low-concentration ATR (0.01 percent) and orthokeratology contact lenses, yielding an annual SER progression and AXL elongation of 0.88 ± 0.31 D and 0.50 ± 0.17 mm [27]. The similar outcome in the DFCL group of the current study may imply that the usage of DFCLs is simpler compared to the combined treatment but has the same efficiency.

Concerning the demographic data, the DFCL group demonstrated an older age and higher baseline myopia degree compared to the ATR group in the current study. The possible explanation for this unbalanced demographic data between the two groups could be related to the fact that users of DFCLs must be older [26]. Generally, DFCLs are recommended for children older than 9 years old in Taiwan due to the need to remove DFCLs oneself during daily life. In fact, all contact lenses, including orthokeratology contact lenses, are rarely applied to patients aged younger than 9 years old in Taiwan. As a consequence, the much older age in the DFCL group compared to the ATR group in the current study is reasonable and can reflect the real-world conditions in Taiwan. Similarly, older age was correlated to a higher degree of myopia in a previous study [28], and thus, the older DFCL group should also show higher myopia than the ATR group. Importantly, we adjusted the effects of age and initial SER in the multivariable analysis so that the imbalance in demographic data would not cause significant statistical bias.

There are certain limitations in the current study. Firstly, the retrospective design of the current study diminishes the heterogeneity of our study groups compared to the prospective study, and the differences in age, myopia degree, and AXL of the two groups could cause significant bias. Secondly, the patients in the current study were managed by different ophthalmologists/optometrists in different branches of our clinic. Accordingly, the habits and opinions of each ophthalmologist regarding myopic control tools may influence the parents’ choice of myopic control tool, and the different autorefractors in each branch and the different refraction techniques of each optometrist may diminish the integrity of our results. Accordingly, the prescription of DFCLs and the judgment of ATR application may not have a universal standard. In addition, we cannot ensure all the measurement times were within the duration we proposed, which could lead to some bias. Moreover, the follow-up period in the current study was only one year, which may not be adequate compared to some long-term studies [25,26]. Finally, all the participants in the current research were Han Taiwanese, and thus, the external validity of the current study may be reduced. Still, because the genotype and phenotype of Han Taiwanese people are similar to the Chinese people who live in mainland China and several other countries, including America, Singapore, and Canada, the results of the current study can be used as a reference for at least 1.5 billion of the population; thus, the clinical relevance of the current study might not be low.

## 5. Conclusions

In conclusion, the application of DFCLs showed a similar effect on myopic control concerning cycloplegic SER progression compared to the usage of high-concentration ATR. Furthermore, the suppression of AXL elongation was similar between the DFCL and high-concentration ATR groups in those without moderate astigmatism and higher AXL. Consequently, DFCLs could be recommended to those with low astigmatism and AXL, considering the lower number of side effects. A further large-scale prospective study to survey the effect of combined DFCL and high-concentration ATR usage on myopic control is mandatory.

## Figures and Tables

**Table 1:** Basic characteristics of the two groups.

Characteristic	ATR Group (N = 182)	DFCL Group (N = 70)	*p* Value
Age	10.26 ± 2.56	12.33 ± 2.09	<0.001 *
Sex (male:female)	45:46	15:20	0.587
Pre-treatment BCVA (LogMAR)	0.004 ± 0.007	0.008 ± 0.004	0.412
Pre-treatment sphere (D)	−1.01 ± 1.42	−1.43 ± 1.47	0.028 *
Pre-treatment cylinder (D)	−0.52 ± 0.60	−0.57 ± 0.49	0.840

ATR: atropine, BCVA: best-corrected visual acuity, D: diopter, DFCL: dual-focus contact lenses, N: number, SER: spherical equivalent refraction. * denotes significant difference between groups.

**Table 2:** The changes in cycloplegic spherical equivalent and axial length between the two groups after the follow-up period.

Outcome	ATR Group (N = 182)	DFCL Group (N = 70)	*p* Value
SER (D)			
Pre-treatment	−1.28 ± 1.41	−1.71 ± 1.56	0.009 *
Post-treatment	−1.59 ± 1.40	−2.03 ± 1.79	0.007 *
Change	−0.31 ± 0.87	−0.32 ± 0.89	0.884
AXL (mm)			
Pre-treatment	24.06 ± 0.72	24.34 ± 0.92	<0.001 *
Post-treatment	24.10 ± 0.78	24.53 ± 0.96	0.007 *
Change	0.04 ± 0.39	0.19 ± 0.26	0.002 *

ATR: atropine, AXL: axial length, D: diopter, DFCL: dual-focus contact lenses, N: number, SER: spherical equivalent refraction. * denotes significant difference between groups.

**Table 3:** Subgroup analysis for the risk of myopia progression in the DFCL group compared to the ATR group with different characteristics.

Subgroup	aOR ^#^	95%CI	*p* Value
SER			
Moderate myopia	1.008	0.420–1.944	0.778
Moderate astigmatism	0.975	0.126–1.875	0.650
High AXL	1.339	0.638–2.588	0.561
Old age	1.015	0.213–2.487	0.723
AXL			
Moderate myopia	1.064	0.847–1.280	0.540
Moderate astigmatism	1.893	1.252–2.557	<0.001 *
High AXL	1.994	1.568–2.817	<0.001 *
Old age	1.472	0.862–2.514	0.157

aOR: adjusted odds ratio, AXL: axial length, CI: confidence interval, SER: spherical equivalent refraction. * denotes significant difference between groups. ^#^ aOR means the DFCL group compared to the ATR group, adjusted for age, sex, pre-treatment BCVA, pre-treatment cycloplegic SE, and pre-treatment AXL.

**Table 4:** The changes in myopic progression parameters between the current study and previous publications.

Parameters	Current Study	Previous Studies
SER progression (D)		
ATR	−0.31	−0.87
DFCL	−0.32	−0.51–−0.52
AXL elongation (mm)		
ATR	0.04	0.23–0.35
DFCL	0.19	0.23–0.30

ATR: atropine AXL: axial length, D: diopter, DFCL: dual-focus contact lenses, SER: spherical equivalent refraction.

## Data Availability

Data are contained within the article.
